# Unraveling the importance of molecules of natural origin in antifungal drug development through targeting ergosterol biosynthesis pathway

**Published:** 2019-12

**Authors:** Fatemehsadat Jamzivar, Masoomeh Shams-Ghahfarokhi, Mansoor Khoramizadeh, Niloufar Yousefi, Mohammadhassan Gholami-Shabani, Mehdi Razzaghi-Abyaneh

**Affiliations:** 1 Department of Mycology, Pasteur Institute of Iran, Tehran, Iran; 2 Department of Mycology, Faculty of Medical Sciences, Tarbiat Modares University, Tehran, Iran; 3 School of Iranian Traditional Medicine, Iran University of Medical Sciences, Tehran, Iran

**Keywords:** Ergosterol biosynthesis, Antifungal drug discovery, Natural compounds, Fungal infections

## Abstract

Over the past decades, the incidence of life-threatening fungal infections has increased dramatically in particular among patients with hampered immune function. Fungal infections cause around 1.5 million deaths annually, superior to malaria and tuberculosis. With respect to high toxicity, narrow spectrum of activity and drug resistance to current antifungals, there is an urgent need to discover novel leads from molecules of natural origin especially those derived from plants and microorganisms for antifungal drug discovery. Among antifungal drugs introduced into the clinic, those affecting ergosterol biosynthesis are still superior to other classes and the vital role of ergosterol in fungal growth and development. This review highlights current knowledge about available antifungal agents and further issues on antifungal drug discovery from compounds of natural origin which affect ergosterol biosynthesis. Special attention is made to the fungal sterol C24-methyltransferase (SMT), a crucial enzyme in ergosterol biosynthesis pathway as a novel target for rational drug design.

## INTRODUCTION

**Fig. 1.**
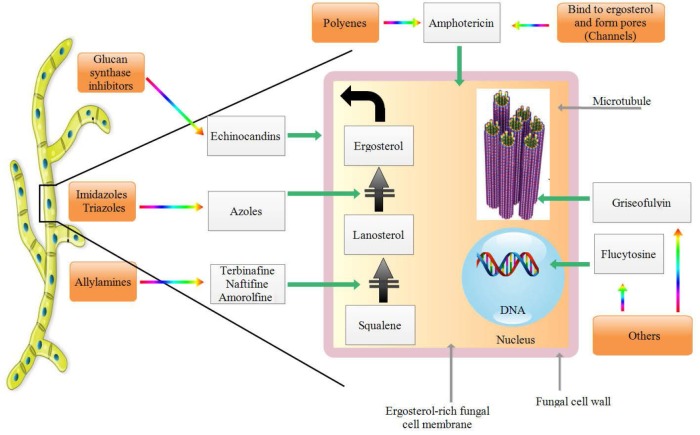
Mode of action of main categories of current antifungal drugs

Nowadays, the mortality rate of fungal infections in the world is more than 1,500,000 cases every year. This figure is higher than mortality due to HIV and malaria infections and equal to tuberculosis. The incidence of infections varies from person to person, and depending on each individual’s immune system, the level of exposure varies considerably (1, 2). Therapeutic methods for invasive fungal infections that are currently in use are very limited in comparison with bacterial infections. Moreover, there are no reliable methods for the treatment of many fungal diseases such as candidiasis, due to the resistance of the etiologic fungi to available antifungal drugs (2). With respect to the high prevalence of fungal infections and the treatment problems associated with such infections, further efforts are needed to identify and detect new antifungal drugs. There are currently three antifungal drug groups used in clinic including polyenes, azoles and echinocandins (3). These antifungals target cell membrane and cell wall components of moulds and yeasts while they have limitations in treatment of invasive fungal infections that has forced researchers to make further studies on the production of new drugs (4) (Fig. 1). For example, famous polyene i.e. amphotericin B has the least function in the safe form of liposome, azoles produce drug resistance and echinocandins prescribed only intravenously are very expensive (4).

With respect to these issues, a series of specific natural compounds have recently been used to produce new antifungal drugs (4). Many of these alternative new natural antifungal drugs are not categorized as antifungal drugs, but alone or synergically, their antifungal effects have been proven (5). The α-bisabolol in chamomile interferes with zymosterol synthesis by a novel mode of action as inhibiting 24-methyl transferase and prevents the foundation of fecosterol from zymosterol in ergosterol biosynthesis (6). Many anti-fungal drugs have natural origin as phenolic acids, flavonoids, tannins, stilbenescurcuminoids, coumarins, lignans, and quinines. They are produced by the plants during secondary metabolism or when a plant is injured. Microorganisms also have the ability to synthesize various groups of natural antifungal compounds (7).

### Challenges and new approaches of antifungal drug development.

Nowadays, the development of new antifungal drugs has over growing increasing. Compared to the advances in new antibiotics that are used to treat bacterial infections, fundamental advances in fungal treatments are challenging due to the eukaryotic nature of fungi (2). One of these challenges is the toxic effect of antifungal drugs in addition to the pathogen fungus on the host. Thus, the three main classes of antifungal drugs are designed to be unique to fungi, and the biggest challenge in the development of new drugs is the complexity of knowing the clinical effects of these drugs (8). Other factors slowing the development of new drugs are lack of sufficient scientific documentation, economic challenges and rigorous monitoring of centers and government agencies (9). Consequently, new creative solutions are needed to overcome these factors.

It has been shown that new antifungal drugs can be produced by targeting ergosterol biosynthesis at the cell membrane as well as targeting the cell wall components of the pathogenic fungi (10). In fact, the mechanism of action of these drugs is to destroy or disable the enzymes or compounds necessary for the survival of the fungi that are present in their cell membrane or cell wall. One of the new classes of antifungal drugs is enfumafungin that is a natural suppressant of GS1 (3-β glucan synthesis) produced by an endophytic *Hormonema* species (11).

**Fig. 2.**
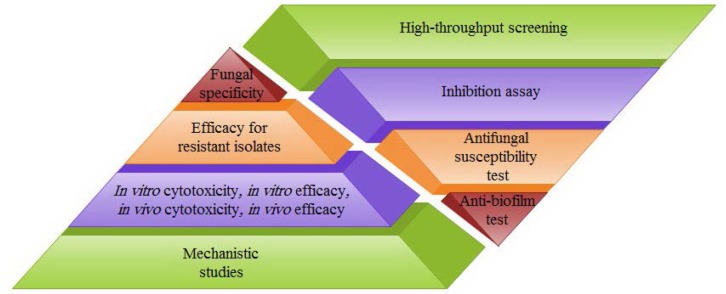
Screening assay for introducing small molecules as novel candidates of antifungal therapy

For the development of new antifungal drugs, finding specific compounds from natural origin by high throughput screening is in progress (Fig. 2). One approach is screening of chemical compounds, in which they are used to create mutations in pathogenic fungi and the resistance to infection in a mutated fungus is evaluated using the wild type of the desired fungus. Use of assessing the lack of growth by increasing the sensitivity or specificity of the combination is another important approach of antifungal drug discovery from natural sources (12, 13).

### Natural product-based antifungal drug discovery.

**Fig. 3.**
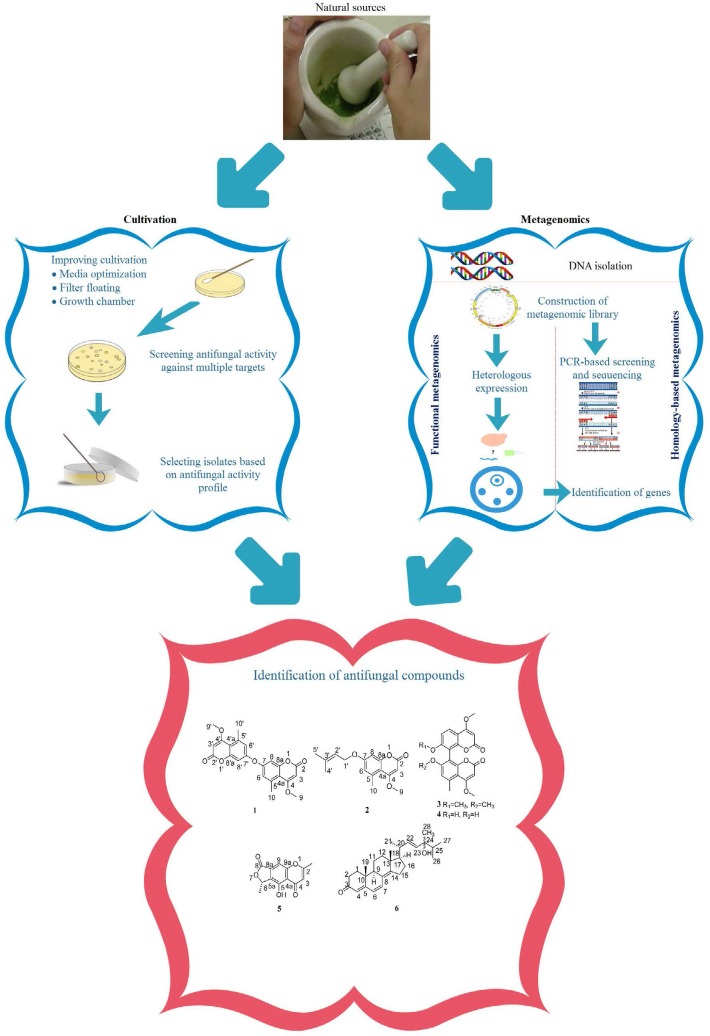
Drug discovery from natural sources: comparison of routine cultivation with metagenomics approaches

Since the discovery of penicillin, the pharmaceutical industry has begun tremendous efforts to use natural compounds to make antibiotic drugs especially in the production of antifungal drugs. Natural compounds that inhibit cell wall synthesis are an important class of antifungal drugs (4). As shown in Fig. 3. antifungal drugs with the natural combination origin, belong to two groups: a group that has a completely natural origin directly extracted from plants or microorganisms through cultivation (5, 14, 15) and another group discovered by using metagenomics approaches.

**Fig. 4.**
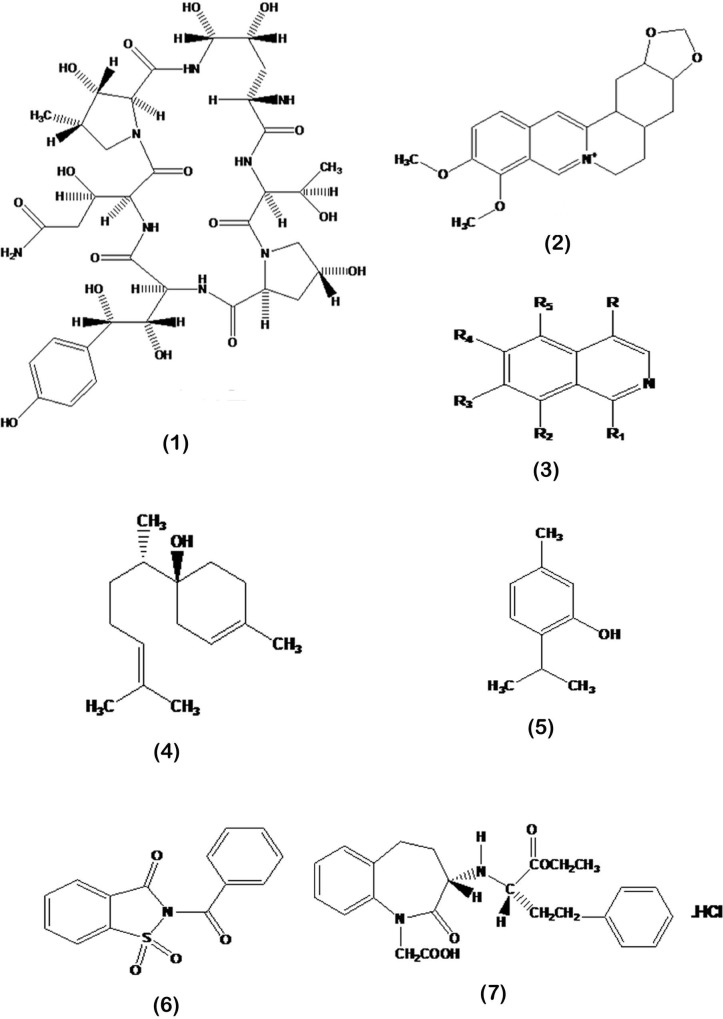
Chemical structure of new antifungal compounds of natural origin

Methods to investigate the genomic structure of natural compounds are also used to determine the best performance of drugs and the best fungi for genomic testing of drugs include: *Saccharomyces cerevisiae, C. albicans* and *Aspergillus fumigatus* (16). Despite the large number of antifungal compounds introduced in recent years, there are very limited reports on the mode of action of such antifungals. Fig. 4. shows the chemical structure of natural antifungal compounds with known mechanisms of action of which echinocandins are selected examples of currently developed antifungals used in clinics. The other compounds are candidates to develop as novel antifungal drugs.

### Echinocandins.

Echinocandins including caspofungin Fig. 4(1), micafungin, and anidulafungin are a new class of antifungal drugs that inhibit the glucan synthesis in the fungal cell wall of main pathogens i.e. *Aspergillus* and *Candida* via inhibiting the enzyme 1, 3-β glucan synthase. As a consequence of β-glucan destruction, resistance against osmotic forces is impaired which leads to fungal cell lysis. It has been shown that echinocandins improve host immune responses via exposing antigenic β-glucan epitopes that trigger host cellular recognition and inflammatory responses.

### Berberine and the isoquinoline alkaloids.

Berberine Fig. 4(2) is a quaternary ammonium salt from the protoberberine group of benzylisoquinoline alkaloids found in various plants as *Berberis vulgaris, Mahonia aquifolium, Hydrastis canadensis, Xanthorhiza simplicissima, Phellodendron amurense, Coptis chinensis, Tinospora cordifolia, Argemone mexicana* and *Eschscholzia californica* (17). The isoquinoline alkaloids Fig. 4(3) are a description of the chemical structures totally named alkaloids (17). Berberine is usually found in the roots, rhizomes, stems, and bark. It has been used synergistically in combination with fluconazole for the treatment of candidiasis due to *C. albicans* in laboratory conditions (17, 18). It has been shown that berberine accumulates in the treated cells causing the cell cycle to stop and reduces transcription in the cell’s genetic cycle (2).

### Antimicrobial peptides (AMPs).

Another group of natural antifungals is antimicrobial peptides (AMPs) and proteins produced by a wide array of biodiversity including plants, fungi, bacteria, insects and humans. This group has a complex mechanism of action. They are potent, broad spectrum antibiotics and demonstrate potential as novel therapeutic agents. They damage the cell membrane, cause apoptosis and cell death, and also impair ion entry and exit in the fungal cell membrane. The modes of action by which antimicrobial peptides kill fungi vary and differ for different fungal species (19). Some antimicrobial peptides kill both fungi and bacteria. The cytoplasmic membrane is mainly affected as a usual target, but peptides may also interfere with DNA and protein synthesis, protein folding, and cell wall synthesis in the fungal cells (20). Likewise, they may penetrate into the fungal cells to bind intracellular molecules which are crucial to cell growth and development. Inhibition of cell wall synthesis, alteration of the cytoplasmic membrane, activation of autolysin, inhibition of DNA, RNA, and protein synthesis, and inhibition of vital enzymes are considered as different modes of action of AMPs. However, in many cases, the exact mechanism of killing of treated fungi is not known (2).

### Bisabolol.

Bisabolol Fig. 4(4) (α-bisabolol or levomenol) is a natural monocyclic sesquiterpene alcohol with sweet aroma soluble in ethanol (21). It is the primary constituent of the essential oil of the plants *Matricaria recutita, Eremanthus erythropappus, Smyrniopsis aucheri, Vanillosmopsis species, Myoporum crassifolium, Arnica longifolia, Aster hesperius* and *Chrysothamnus nauseosus* (6, 22–25). It was shown that *Vanillosmopsis* sp, *M. chamomilla* and *S. runcinata* contain 50 and 90% α-bisabolol (26, 27). It has been shown that α-bisabolol in *Candeia* wood (*Eremanthus erythropappus*), may contain 85% α-bisabolol (28). α-bisabolol has been introduced as generally regarded as safe for human health by the Food and Drug Administration. The main use of α-bisabolol in pharmacy is related to the production of anti-inflammatory, anti-spasmodic and anti-allergic drugs (28, 29). Bisabolol is known to have anti-irritant, anti-inflammatory, and antifungal and antimicrobial properties (24, 30, 31).

α-bisabolol induces apoptosis by decreasing oxygen consumption in human and mouse glioma cells via disturbing the structure and function of the mitochondrial permeability transition pore (32). It has strong antifungal activity toward different pathogenic fungi (6, 33, 34). It has been proposed that α-bisabolol also induces apoptosis in fungi by interacting with fungal hyphal membranes.

### Phenolic compounds.

Many antifungal compounds have phenolic origin. They are produced by the plants during secondary metabolism or when the plant is injured. Microorganisms also have the ability to synthesize phenolics (7). Phenolic acids, flavonoids, tannins, stilbenescurcuminoids, coumarins, lignans, quinines are some examples of natural compounds with phenolic origin adopted from the pharmaceutical herbs and dietary plants. Curcumin made by *Curcuma longa* has been showed to exert toxic affect against *C. albicans* as well as non-albicans species by enhancing reactive oxygen species (ROS) levels and inducing early apoptosis. It also reported to inhibit *Aspergillus parasiticus* growth and aflatoxin production by the fungus via affecting essential genes in the toxin biosynthetic pathway (35). Ajugol from *Spathodea campanulata* is a phenolic compound of the plant fruit which has been reported to have anti-fungal properties (36). Bisbibenzyl is a new antifungal agent that prevents the growth of *C. albicans* by inhibiting the morphogenetic shift and by inhibiting biofilm formation due to up-regulation of *DPP3* gene (37). Phenolics from *Spirulina*, a cyanobacterium has shown to inhibit fungal growth by an ergosterol-suppressive effect (38). 2,5-dihydroxybenzalde-hyde exhibited synergistic effects with itraconazole and amphotericin B against *C. neoformans*. Similarly, thymol Fig. 4(5) also shows synergistic effect with azoles such as fluconazole (39). Thymol can interfere with the fungal growth in two ways; a disturbance in the cell wall integrity (40) or changes in the plasma cell membrane by inhibiting ergosterol biosynthesis (41). Various phenolics such as salicylic acid, phenol and benzoic acid inhibit the growth of *F. oxysporum* by an unknown mechanism (42).

### Benzisothiazolinone compounds (BzT).

By using a development therapeutics program (DTP) library containing multiple and classified compounds, a low molecular weight compound group named benzisothiazolinone compounds was created by several advanced laboratories (3, 43). Scaffolds such as the amino acid-derived 1, 2-benzisothiazolinone Fig. 4(6) scaffold were screened against *Candida* species, *Cryptococcus neoformans, Aspergillus fumigatus* and several dermatophytic fungi (43, 44). These studies revealed that a methyl group and a phenyl ring are required for most fungal activity. All of these compounds causes synergistic effects with fluconazole and with a similar concentration of micafungin and amphotericin B exhibited a fatal effect and had a minimum toxicity for human cells (43).

### Novartis compound archive.

Another method used for the identification of new antifungal compounds is using the Novartis compound archive Fig. 4(7), which is obtained from *S. cerevisiae* by the method of haplo insufficiency profiling (HIP) (45). There are other extraction methods that each of them has different effects on a specific species of fungi (2). All of these methods determined that for the production of antifungal drugs by using the method of HIP, *S. cerevisiae* or the profile of other pathogen fungi can be the most practical way to make new combinations of drugs (2).

### Targeting ergosterol biosynthesis via inhibition of C24-methyltransferase (24-SMT).

Sterol metabolism is one of the main differences between human and pathogens biochemical metabolism, so it can be used to increase the effectiveness of antifungal and anti-parasitic drugs. Fungi and protozoans rather than animals have more similarities and proximity to plants that use of these similarities to synthesis 24-alkyl side chains characteristic of the C28 and C29 membrane inserts ergosterol and sitosterol found in these organisms (46, 47). The enzyme that catalyzes the conversion of Δ^24^-sterol acceptor molecules to 24-alkyl sterols is sterol C24-methyltransferase (24-SMT) (47). This usually prevents and limits the synthesis of phytosterol, and is expressed as a template and a plan to explain the variability and characteristics of sterol. 24-SMT can show its performance in many ways as a single functional (C1-transfer activity) or bifunctional (C1 and C2-transfer activities) enzyme (46, 48). Enzymes are located within the cell’s endoplasmic reticulum and there are two substrates in the cell, one of them is methyl donor AdoMet and the other one is Δ^24^-sterol acceptor, in the construction of the phytosterol side chain.

The 24-SMT is not synthesized in animals, making it a novel target for logical medicines design. When 24 alkylsterol (ergosterol) was converted into a balanced state of 24-desalkyl sterols (lanosterol / zymosterol). It is seen that the end path of sterol production is expressed as an agent in the formation of undesirable steps in the structure of ergosterol (49, 50).

Substances that stop the synthesis of ergosterol are called EBIs (ergosterol biosynthesis inhibitors). They have nitrogen and different circular structures and that are classified according to these circular structures. Some of these substances are used only in agriculture. EBIs that derived from pyridine and pyrimidine are mostly antifungal compounds in the treatment of local dermatophytosis. As shown by various studies, all of them prevent the biosynthesis of ergosterol. The piperazine, pyrimidine, imidazole and triazole types of EBI specifically inhibit the oxidative removal of sterol C (14) methyl groups by the cytochrome P-450 enzyme (51, 52). In the pathway of ergosterol synthesis in *A. fumigatus*, 20 genes are involved, and some of them are in several copies. Although the number of carriers of final codes (ATP-binding-cassette) are similar in all species of *Aspergillus*, but the ABC of fungi that inhibited by several drugs are more (53). Comparative analyses about the ergosterol biosynthesis were done in three species of *Aspergillus* (53). The Erg route is very important because it can be used to enhance inhibitors by increasing activity or a wider range of them. The comparative analysis of this pathway in three distinct *Aspergillus* species showed that there are several copied genes in this pathway. It is interesting that, *erg3* showed two, three, and four copies in *A. nidulans, A. fumigatus* and *A. oryzae*, respectively, while *erg11* presented two copies in *A. nidulans* and *A. fumigatus*, and three copies in *A. oryzae* (53). Besides, if a change in the precursors of the ergosterol biosynthesis pathway occurs, its production will be stopped. For example, in *S. cerevisiae*, if a change occurs in the stages of ergosterol production, it leads to the production of lanosterol instead of ergosterol (54). Study of sterol changes by GC-MS and H1NMR methods showed that C-methylation of this reaction by 24-C-methyltransferase (24-SMT) takes place.

General studies have shown that there is no base substrate for inhibiting 24-SMT in humans and for antifungal treatment; ergosterol homeostasis can be used to design antifungal drugs. The way that homeostasis is present in ergosterol is different from how drugs work, for example, the polyene antibiotic binds to ergosterol in the membrane fats and causing interference in the permeability of the cell membrane, while EBIs effect on the target enzyme and causing a breakdown of carbon flow that is needed for ergosterol and accumulation of mediators within the cell. These compounds act at the lowest levels of their concentration in the 14α-demethylation phase of the lanosterol metabolism. This step is controlled by the CYP51 enzyme that is identical in fungi and humans (55, 56). C14-demethylation most of them has a dedicated and unit site. In recent years, concerns have been raised for their resistance, which with different methods such as mutation in the main structure of the compound; it is possible to produce non-resistive drugs (57, 58).

24-SMT of the wild type of *C. albicans* has been extensively studied (59). The external metabolism that prepares Δ^24^-sterols for giving ergosterol and also the observed properties for it *in vitro*, showed that in yeast 24-SMT is as a receptor and in the form of combination with C-24 alkylation sterol. This may be due to the lateral chain building and the specific physiology of ergosterol. This feature can be used as a goal for the development of chemical therapies methods (59).

To determine what specific structural features of the C3-polar group ensure sterol catalysis, a series of structurally related C3-analogs of lanosterol that differed in stereochemistry, bulk and electronic properties were examined against the fungal 24-SMT from *Paracoccidioides brasiliensis* (Pb) which recognize lanosterol as the natural substrate. Researchers studied the magnitude of sterol C24-methylation activity (based on the kinetic constants of Vmax/Km and product distributions determined by GC–MS (60). Resulting from changes at the C3-position in which the 3β-OH was replaced by 3α-OH, 3β-acetyl, 3-oxo, 3-OMe, 3β-F, 3β-NH2 (protonated species) or 3H group revealed that lanosterol and five substrate analogs were catalyzed and yielded identical side chain products whereas neither the 3H- or 3α-OH lanosterol derivatives were productively bound (60)fig.

**Fig. 5.**
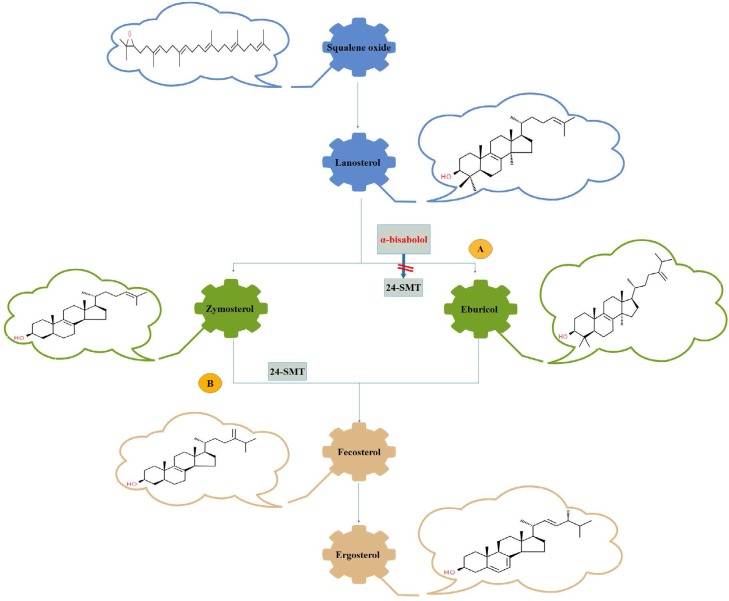
The mechanism of action 24-SMT: Inhibition of ergostrol biosyntetise with the enzyme 24-SMT occurs in several forms

Important natural compounds that have a structural similarity between fungi and plants, are terpenoids from aromatic plants, which due their similarity to the 24-SMT enzyme present in both fungi and plants, making them suitable to produce a lot of antifungal drugs like α-bisabolol and put them into the market. Pauli (33) proposed that α-bisabolol inhibits fungal growth by affecting ergosterol biosynthesis. However, he did not introduce any obvious document for his claim. Jahanshiri et al. (6) showed that α-bisabolol strongly inhibited *Aspergillus fumigatus* growth (35.53–77.17%) and ergosterol synthesis (26.31–73.77%) dose-dependently and suppressed the expression of *erg6* gene by 76.14% at the highest concentration of 9 mM. They clarified that α-bisabolol inhibits the activity of 24-SMT by 99% at the concentration of 5 mM. As shown in Fig. 5, these results provided evidence for the first time that α-bisabolol inhibits *A. fumigatus* Af239 growth via affecting microsomal Δ^24^-sterol methyltransferase as a crucial enzyme in ergosterol biosynthetic pathway. This finding is surprising because shows that novel antifungal compounds may target ergosterol biosynthesis in specific action sites such as 24-SMT which is absent in mammalian cells.

## CONCLUDING REMARKS AND FUTURE PROSPECTS

The development and production of antifungal drugs has progressed markedly since the 1950s with the spread of cryptococcosis disease in the world, at that time penicillin was the only known drug that used for all infections. When amphotericin was discovered and developed, fungal treatments improved significantly. At present, the growth trend of novel antifungal drugs is very slow compared to the rise in fungal infections. The reason for this is the low demand of market, low profits, and the opinion of drug manufacturing companies that the use of these new drugs is more expensive than current drugs. Therefore, it is quite logical to research about new anti-fungal drug compounds based on natural compounds and originated from nature at reasonable prices. This review further indicates that broad spectrum bioactive molecules by natural origin which target specific sites in the ergosterol biosynthesis pathway such as α-bisabolol are potential candidates for drug development against a wide array of fungi with least toxicity for the mammalian host. Further studies for drug discovery based on omics approaches are quite necessary to reduce the upcoming challenges in making new drugs.
